# Effect of folate deficiency on promoter methylation and gene expression of *Esr1, Cav1*, and *Elavl1*, and its influence on spermatogenesis

**DOI:** 10.18632/oncotarget.15731

**Published:** 2017-02-25

**Authors:** Hong-Fang Yuan, Kai Zhao, Yu Zang, Chun-Yan Liu, Zhi-Yong Hu, Jia-Jing Wei, Ting Zhou, Ying Li, Hui-Ping Zhang

**Affiliations:** ^1^ Family Planning Research Institute, Tongji Medical College, Huazhong University of Science and Technology, Wuhan, China; ^2^ Center of Human Reproduction, Tongji Medical College, Huazhong University of Science and Technology, Wuhan, China; ^3^ Department of Urology, Tongji Hospital, Tongji Medical College, Huazhong University of Science and Technology, Wuhan, China

**Keywords:** folate deficiency, spermatogenesis, methylation, male infertility, gene expression, Pathology Section

## Abstract

This study aims to investigate the effect of folate deficiency on the male reproductive function and the underlying mechanism. A total of 269 screened participants from 421 recruitments were enrolled in this study. An animal model of folate deficiency was constructed. Folate concentration was measured in the ejaculate, and its association with semen parameters was then determined. The expression and promoter methylation status of *ESR1*, *CAV1*, and *ELAVL1* were also evaluated. Results showed that seminal plasma folate level was significantly lower among subjects with azoospermia than those with normozoospermia. Low folate level was significantly correlated with low sperm concentration in men with normozoospermia. Folate deficiency significantly reduced the expression of *ESR1*, *CAV1*, and *ELAVL1*, which are critical to spermatogenesis. However, low folate levels did not increase the methylation levels of the promoter regions of *ESR1*, *CAV1*, and *ELAVL1* in human sperm DNA. Thus, folate deficiency impairs spermatogenesis may partly due to inhibiting the expression of these genes. Thus future research should determine the significance of sufficient folate status in male fertilization and subsequent pregnancy outcomes.

## INTRODUCTION

Infertility is estimated to affect up to 186 million people worldwide [1]. Male factors contribute to approximately 30%-55% of all cases, which are mainly related to impaired spermatogenesis [2]. Spermatogenesis is a highly complex process that involves a multitude of genes whose expression is precisely regulated by a series of choreographed molecular events and signaling pathways [3]. If genes related to the cell division and maturation of spermatids are expressed abnormally, then sperm quality will be significantly affected, leading to infertility [4, 5]. Gene expression is affected not only by macronutrients but also by micronutrients, including folate [6, 7], which must be provided by diet or supplementation. Folate, as the initial substrate in one-carbon metabolism, is converted into 5-methyltetrahydrofolate in liver prior to distribution to various tissues. Subsequently, 5-methyltetrahydrofolate catalyzed by methionine synthase with vitamin B12 as cofactor provides homocysteine with a methyl group to form methionine and S-adenosyl-methionine (SAM), the universal methyl donor required for methylation of DNA, RNA, proteins, and lipids and their methylation maintenance. Moreover, 5-methyltetrahydrofolate catalyzed by methylenetetrahydrofolate reductase (*MTHFR*) generates tetrahydrofolate and 5,10-methylenetetrahydrofolate, which are used for purine and thymidylate biosynthesis, respectively. Despite the essential functions of biosynthesis and methylation, majority of studies have focused on the role of the folate in the normal reproductive function of women. Thus far, the role of folate in in male reproduction, particularly spermatogenesis and spermatogenesis-related gene expression, has been rarely investigated.

Folate plays a vital role in spermatogenesis by deleting key genes involved in folate metabolism pathway; these genes include reduced folate carrier [8] and *MTHFR* [9]. Animal experiments and clinical research indicate that folate deficiency is correlated with decreased sperm concentration [10, 11]. Treatment with antifolate drugs for various malignant tumors leads to poor semen quality [12–14]. Furthermore, randomized clinical trials reported that folic acid supplementation for 6 months significantly improved the semen parameters of varicocelectomized subjects [15] and increased the sperm concentration of subfertile males [16, 17]. Previous studies employed less defined study subjects, simple statistical analysis and small sample size [11, 18, 19]; as such, the correlation between folate status and human semen variables remains unclear. Furthermore, the mechanisms underlying the adverse effect of folate deficiency on spermatogenesis have not been elucidated yet.

Many key signaling molecules play pivotal roles in regulation of spermatogenesis. The expression of estrogen receptor 1 (*Esr1*), caveolin-1 (*Cav1*), and ELAV-like RNA binding protein 1 (*Elavl1*) shows specific spatial patterns in mouse testis [20–22]. Disturbing the expression of these three genes causes poor semen quality, eventually leading to sterility. The infertility of the established *Esr1*−/− male rats could be attributed to the pronounced structure disorganization of their testes [23]. Moreover, *Elavl1*−/− mice had a complete loss of spermatozoa as a result of germ cell differentiation defects [24]. *Cav1*, a small integral membrane protein, was detected in both mouse and guinea pig sperm cells; this gene was implicated in acrosome biogenesis [25, 26]. Previous studies demonstrated that folate deficiency affected the gene expression of *Esr1*, *Cav1*, and *Elavl1* [10, 27, 28]. However, whether or not impaired spermatogenesis related to insufficient folate is caused by inhibiting the expression of these three genes remains unknown.

The total folate concentration in seminal plasma is approximately 1.5 times higher than that in blood [11, 16, 17]. A positive relationship exists between seminal fluid folate and blood plasma folate [11], implying that folate level in seminal plasma may reflect the overall folate nutriture.

This study aims to explore the effects of folate deficiency on male reproduction. Folate level in the ejaculate was measured, and the associations between folate concentration and semen parameters were determined. The expression and promoter methylation status of *Esr1*, *Cav1*, and *Elavl1* were then assessed using sperm samples and an animal model.

## RESULTS

### General characteristics and semen parameters of screened subjects

A total of 269 participants, including 71 patients with azoospermia and 198 with normozoospermia, were enrolled in this study. Table 1 summarizes the general characteristics and semen variables of the screened subjects. The median age of all study subjects was 31 years old (range, 23-51); the median value of BMI was 22.0 (range, 17.2-29.7); and the median abstinence time was 5 days (range, 2-7). Men with normozoospermia were older than those with azoospermia, but the difference in age was not significant. However, the semen volume of subjects with azoospermia was smaller than that of normozoospermic men (*p* < 0.01). Among those with azoospermia, five males exhibited semen pH < 7.2 (7%).

**Table 1:** Selected characteristics of the study population

Characteristic	Azoospermia (n=71)^a^	Normozoospermia (n=198 )^a^	*P*-value^b^
Demographic			
Age (y)	30 (23—49)	31 (24—51)	0.06
BMI (kg/m^2^)	21.3 (17.3—29.7)	22.1 (17.2—29.4)	0.23
Semen parameters			
Ejaculate volume (mL)	2.9 (0.7—6.1)	3.5 (1.5—10.9)	<0.01
Sperm density (10^6^/mL)	—	71.50 (15.67—388.60)	—
Sperm count (x 10^6^)	—	255.34 (39.46—1998.09)	—
Sperm progressive motility (%)	—	46.55 (32.01—81.57)	—
Sperm normal morphology (%)	—	43.79 (19.27—71.15)	—
PH	7.4 (6.0—7.4)	7.4 (7.2—7.5)	0.00
Duration of abstention (d)	4 (2—7)	5 (2—7)	0.24

^a^All variables were presented as median (range).

^b^Differences between Azoospermia and subfertile men were analyzed using the two-tailed Mann-Whitney U test.

Abbreviation: BMI: Body Mass Idex.

### Folate level in seminal plasma and its relationship to semen parameters

**Figure 1:**
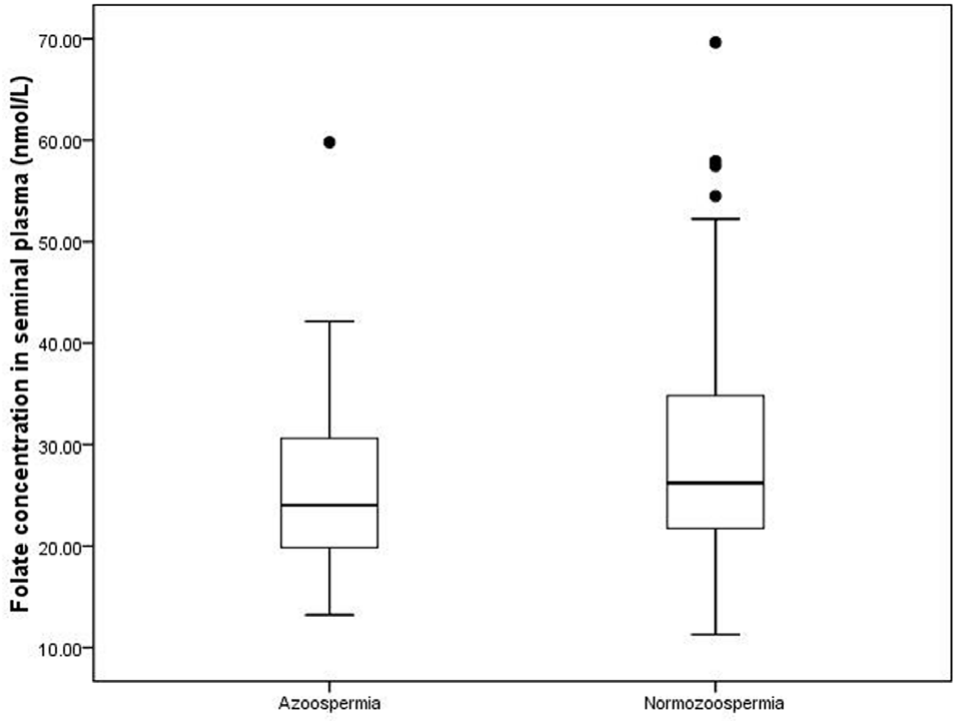
Distribution of folate level in seminal plasma in two groups Box plots of seminal plasma folate concentrations in the 269 study subjects. In each plot, the solid horizontal lines mark the 10th, 25th, 50th, 75th and 90th percentile points of the data. The box encompasses the 25th through the 75th percentiles.

For all the subjects, the median seminal plasma folate was 25.79 nmol/L, with a range of 11.27 and 69.64 nmol/L. Figure 1 shows the distributions of folate concentrations in the seminal fluid of subjects with azoospermia and normozoospermia. The median seminal plasma folate concentration in subjects with azoospermia was 24.01 nmol/L, with an interquartile (25th and 75th percentile) range of 19.84 and 30.69 nmol/L. By contrast, the median concentration of seminal plasma folate in subjects with normozoospermia (26.21 nmol/L) is statistically significantly higher, with wider interquartile range (21.73-34.83 nmol/L), than that in subjects with azoospermia. Table 2 depicts the correlation between the seminal plasma folate and semen parameters in subjects with normospermia. The seminal plasma folate concentration was statistically significantly correlated (r = 0.19, *P* < 0.01) with semen density but not with other semen variables. After adjusting for possible confounders (age, BMI, and abstinence duration) by using multiple linear regression analysis, the aforementioned significant correlation (*P* = 0.01) still existed.

**Table 2:** Correlation of folate in seminal plasma and semen parameters of 198 participants #

Semen parameters	volume	density	Total count	Progressive motility	Normal morphology
Folate concentration	-0.11	0.19^**^	0.13	-0.12	0.09

^#^Correlation was determined by Spearman rank correlation coefficients.

^**^P < 0.01.

### Effects of folate deficiency on sperm production

**Figure 2:**
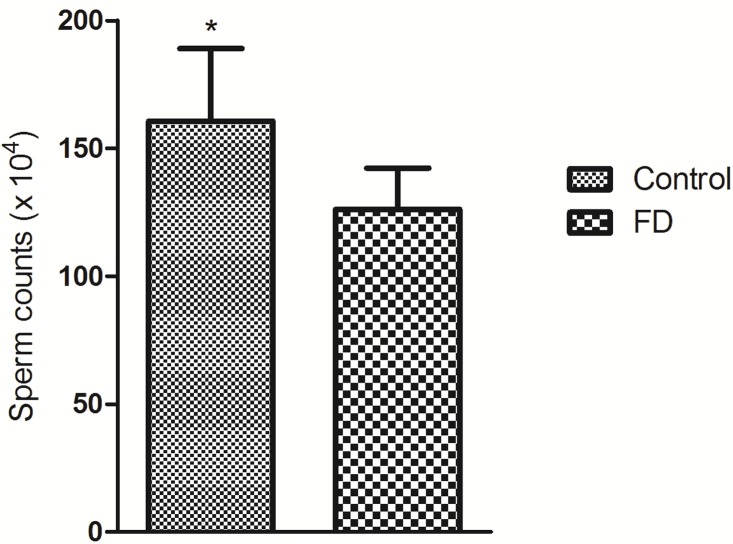
Effect of folate deficiency on sperm counts in mice Means ± SD of determinations (each group, *n* = 6) are shown. ^*^P < 0.05 by Student's *t*-test.

**Figure 3:**
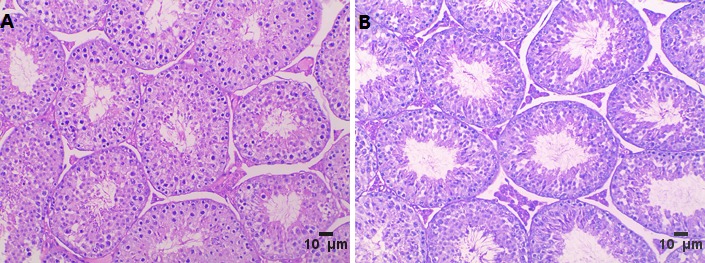
Haematoxilin/eosin staining **A**. control group, **B**. folate deficiency group, Scale bars, 10 μm.

To study the effect of inadequate folate on male reproduction function, we constructed an animal model with folate deficiency (FD) diet as described in a previous study [10]. The total plasma folate levels (6.11 ± 1.83 μmol/L, n = 6) in the FD group were significantly lower than those in the control group (13.83 ± 4.35 μmol/L, n = 6; *P* = 0.002). This result confirmed the effectiveness of the FD diet treatment. Compared with the regular rodent chow, the FD diet did not affect testis and body weights (data not shown). However, the sperm count slightly significant decreased in FD males than that in control males (*P* = 0.049) (Figure 2). Histological examinations of adult testis (8 weeks) were performed to identify pathological changes (Figure 3). Figure 3A shows that mice in the control group exhibited a normal testicular morphology with regular germ cell arrangement. The experimental group treated with FD diet exhibited focal necrosis of cells lining the seminiferous tubules along with cellular disorganization (Figure 3B).

### Changes in the expression of *Esr1*, Cav1, and *Elavl1*

**Figure 4:**
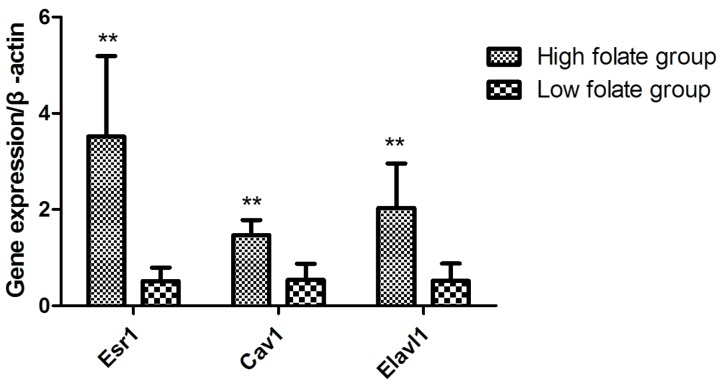
Effect of low folate level in human seminal fluid on gene expression of *Esr1*, *Cav1* and *Elavl1*, ^**^P < 0.01

**Figure 5:**
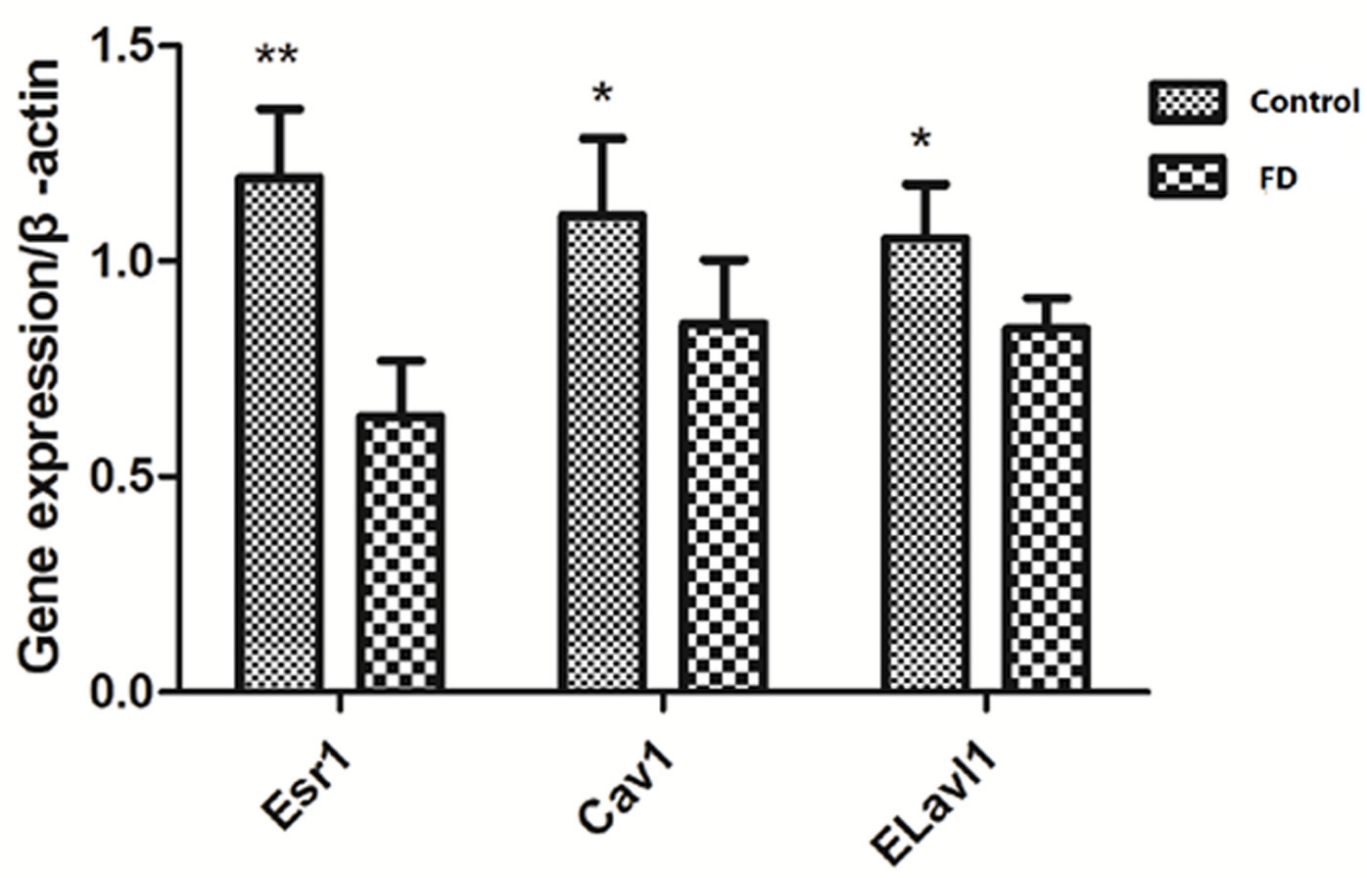
Effect of low dietary folate on gene expression of *Esr1*, *Cav1* and *Elavl1* of mice, FD: folate deficiency, **P < 0.01, *P < 0.05

**Figure 6:**
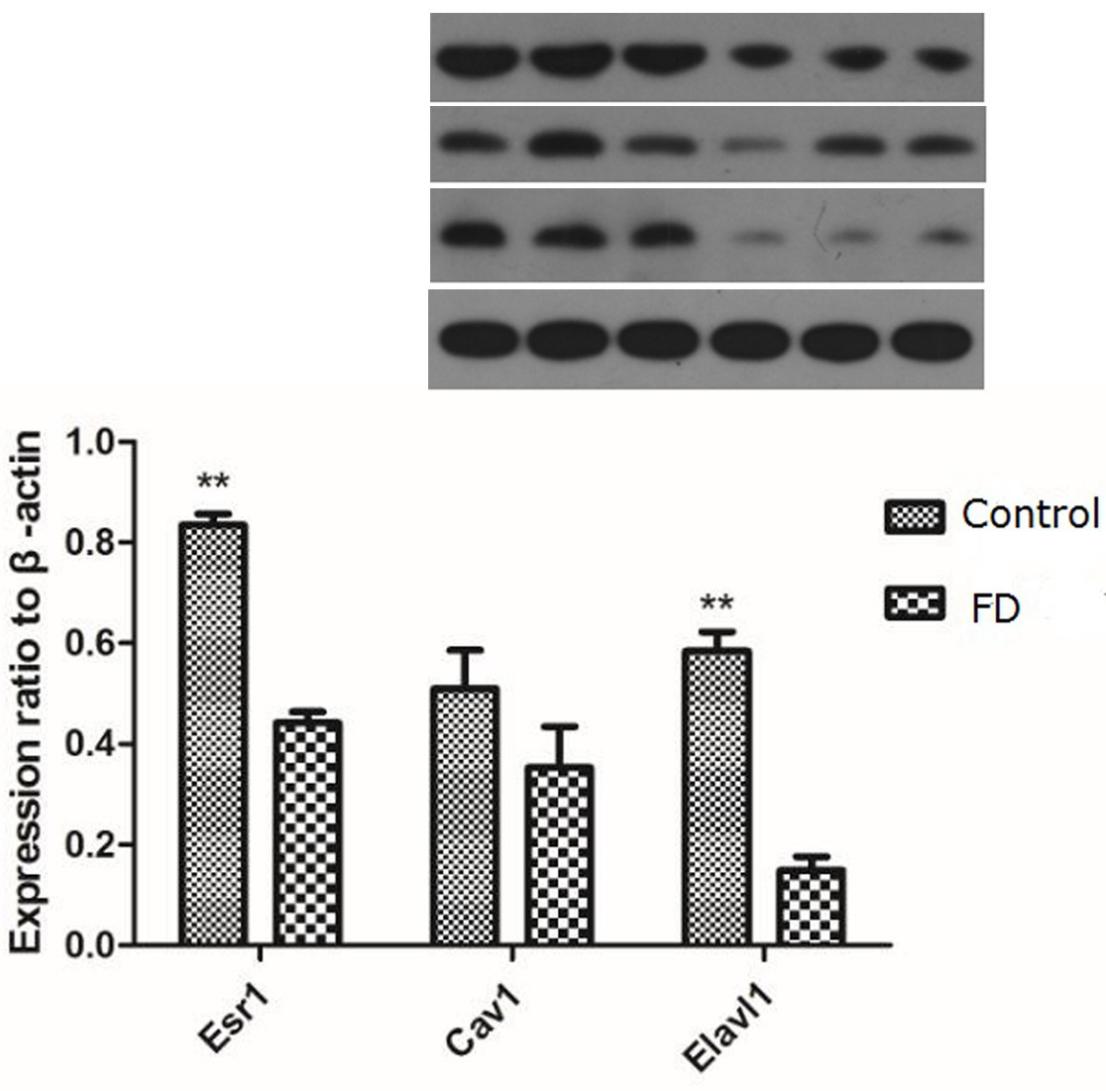
Effect of low dietary folate on Expression levels of *Esr1*, *Cav1* and *Elavl1* proteins in each mice group, FD: folate deficiency, **P < 0.01

Considering that *Esr1*, *Cav1*, and *Elavl1* are involved in regulating sperm production and maturation, we used human sperm samples and constructed a mouse model to investigate the effects of folate deficiency on gene expression. With regard to the human sperm samples (as shown in Supplemental Table S1), the mRNA levels of *ESR1, CAV1* and *ELAVL1* in the group with low seminal plasma folate concentration are significantly lower than those in the group with high seminal plasma folate concentration (*p* < 0.01, Figure 4). To further study the effects of folate deficiency, we detected the expression of these genes in testes sample of the mouse model through RT-qPCR and Western blot analyses. As shown in Figure 5, the relative mRNA levels of *Esr1*, *Cav1*, and *Elavl1* in the folate-deficient mice (FD group) were also lower than those in the control group (*p* < 0.001, *p* = 0.031, and *p* = 0.001, respectively). The protein expression levels of *Esr1* and *Elavl1* significantly decreased in the FD group compared with that in the control group (*p* < 0.01). Although the protein expression level of *Cav1* was low in FD mice there was no significant different compared to control mice (Figure 6).

### Methylation profile of the *Esr1*, Cav1, and *Elavl1* promoter regions in human sperm

**Figure 7:**
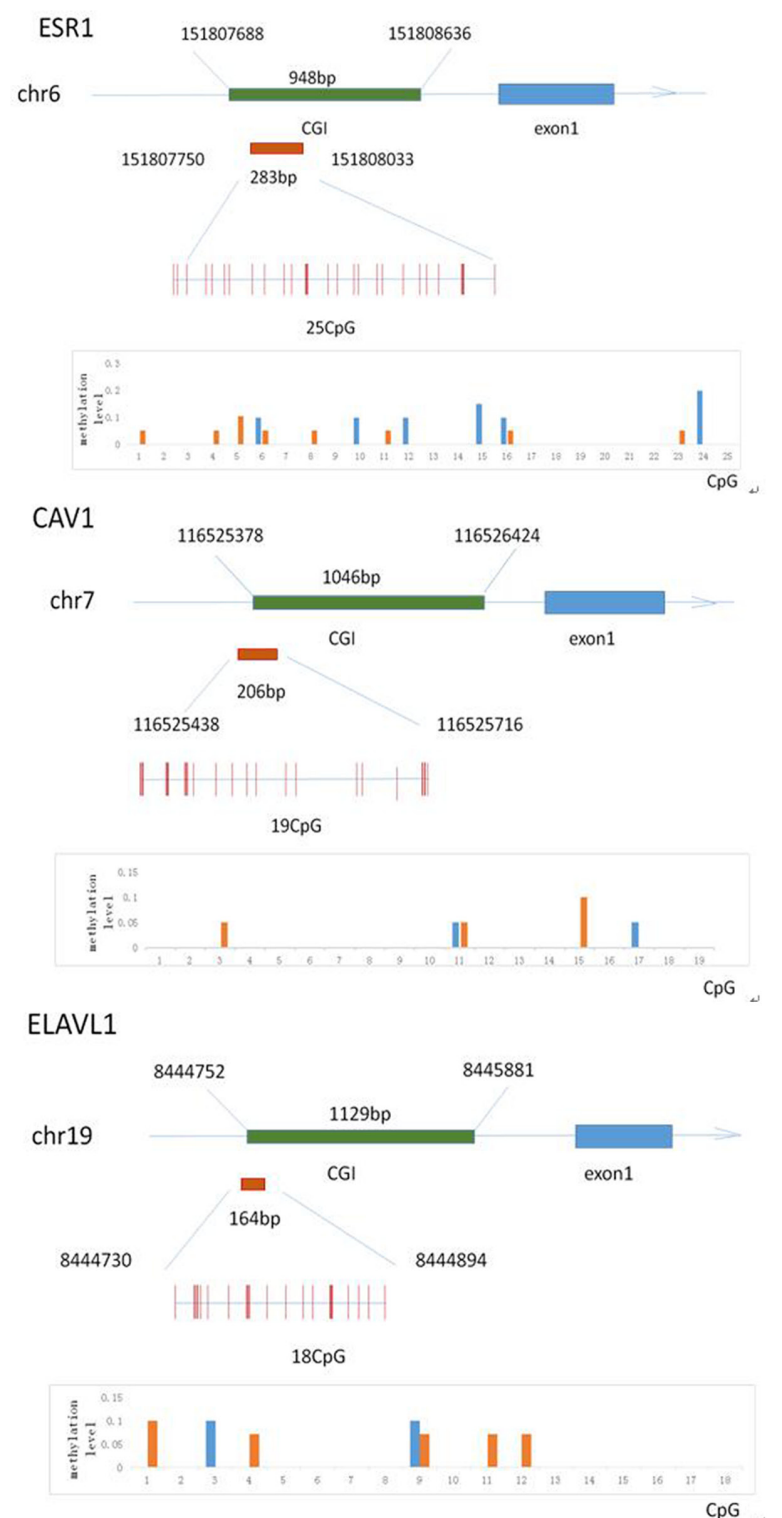
The methylation status of the *Esr1*, *Cav1* and *Elavl1* promoter region in human sperm sample Schematic diagram **A**., **B**. and **C**. indicates the classic 25 (*Esr1), 19*(*Cav1*) and 18 (*Elavl1*) CpG sites in the 5′-flanking region, respectively. Red columns represent CpG sites. Graphical representation of data from subjects with low folate and high folate level in seminal plasma, respectively, showing distribution of methylated CpGs in two groups.

To investigate the effect of folate deficiency on the methylation status of the *ESR1, CAV1* and *ELAVL1* promoters, we selected 20 semen samples with low ( < 25th percentile) and high (> 75th percentile) folate concentrations. Supplemental Table S1 shows information about the two groups. To precisely examine the methylation frequency, we used BSP for quantitative assessment of the methylation status of each CpG site in the *ESR1, CAV1* and *ELAVL1* promoters (Figure 7). We randomly selected four clones of the promoter of each gene from each sample for analysis of the CpG islands in each group. Twenty-five *ESR1* CpG sites, nineteen *CAV1* CpG sites, and eighteen *ELAVL1* CpG sites were detected. The methylated CpG sites of *ESR1, CAV1* and *ELAVL1* in the sample with low seminal plasma folate levels were 5.5%, 2.5%, and 3.0%, respectively, whereas those in the sample with high folate levels were 4.5%, 3.5%, and 3.0%, respectively (P > 0.05). The overall methylation levels within these promoter sequences were no significance between 2 groups. Thus, folate deficiency did not significantly affect the methylation frequency of the *ESR1, CAV1 and ELAVL1*promoters.

## DISCUSSION

Although folate deficiency adversely affects human reproduction, few studies have investigated its effects on male reproductive health. In the present study, low folate levels in seminal plasma may contribute to impaired spermatogenesis and are significantly correlated with low sperm concentration in men with normozoospermia. In addition, folate deficiency can evidently reduce the gene expression levels of *ESR1*, *CAV1* and *ELAVL1*, which are critical to spermatogenesis. However, low amounts of folate do not affect the methylation profile of the *ESR1*, *CAV1* and *ELAVL1* promoter regions in human sperm DNA. To our best knowledge, this study is the first to report the effect of folate deficiency on male reproductive function by using the combination of human research and respective animal study.

The total folate concentration in seminal plasma of men with azoospermia is significantly lower than that of patient with normozoospermia; hence, sufficient folate levels may be required for normal spermatogenesis. However, Igor et al. [29] demonstrated that the median levels of seminal plasma folate is lower among men with azoospermia than those among those with normozoospermia, but the difference is not significant. The discrepancy in the results could be due to the differences in the demographic characteristics of the subjects, sample study size and detection technology. The median value of the seminal plasma folate level in Chinese adults is higher than that in people in the United States [11] but is similar to that of people in the Netherlands [18]. The positive relationship between folate level and sperm concentration is easily understood in view of its fundamental importance in biological function. This correlation was not found in other studies with small sample size [18, 19] but in line with the study of Wallock et al. [11]. Furthermore, analysis using the established folate-deficient animal model showed that absence of access to folate significantly reduced sperm count, similar to the findings of Swayne et al. [30] and Austin et al. [8]. By contrast, Lambrot et al. [10] did not found that folic acid deficiency in early development can alter sperm counts in adult mice. In addition, many studies suggested that polymorphisms in folate-metabolizing genes increased the risk of male infertility mainly caused by folate deficiency [31–33]. Overall, low levels of folic acid negatively affect the male reproductive health.

Although studies have established that folate deficiency diet affected the gene expression pattern in many organs, including the liver, kidney, spleen, and testis, in mice[7, 10, 34], to our knowledge, the present study reported for the first time that folate deficiency altered the amount of several mRNAs in the human sperm. Because of the loss of the transcription ability of mature sperm, the effect of folate deficiency may arise in the early stage of spermatogenesis. The altered mRNA expression profile may affect the pregnancy outcome and offspring health owing to the delivery of spermatozoa RNA [35].

*ESR1* is expressed in Leydig cells, epithelium of efferent ductules, and some germ cells, particularly spermatocytes and spermatids [36]. In comparison with the wild-type mouse testes, *Esr1* knockout mouse testes displayed atrophied seminiferous epithelia caused by the disruption of some critical signaling pathways, such as MAPK3/1 phosphorylation [37, 38]. Gao et al. [28] reported that folate deficiency decreased Esr1 gene expression in the endometrium, which is in line with the results from the present animal research. Similarly, our data confirmed the reduced *Cav1* gene expression in folate-deficient sired mouse, as revealed by the microarray analysis [10]. *CAV1*, the major component of caveolae, contributes to acrosome biogenesis [25, 26] and repairs DNA damage [39]; hence, *CAV1* is closely correlated with normal sperm function. Evidence shows that *Elavl1*, affects DNA methylation patterns by stabilizing and increasing the levels of *DNMT3b* mRNA [40]. Nevertheless, limited information is known about the unavailability of folate on gene *Elavl1*. Insufficient folate levels can lead to high homocysteine concentration. Hyperhomocysteine may inhibit the enzyme activities during transcription and translation to reduce gene expression. So we speculated that the negative effects of folate deficiency on male reproductive function may be due to their abnormal expression.

Low levels of dietary folate cause aberrant DNA methylation pattern and can lead to perturbed biosynthesis [10, 41]. Therefore, we determined the methylation profile of the *Esr1*, *Cav1*, and *Elavl1* promoter regions in the human sperm DNA. We found that the methylation status in the promoter regions of the abovementioned three genes was not altered by the folate level. Moreover, DNA methylation did not directly affect the corresponding gene expression. Interestingly, previous studies reported similar findings in the animal model [28, 42]. Conversely, other studies showed the altered methylation profile in the promoter regions [43]. We speculate that some genes are more sensitive to folate deprivation than others.

Our study had several limitations and strengths. First, the polymorphisms in the folate-related enzyme genes were not detected. In addition, a recent study did not find an association between the variants of three common folate metabolic enzyme genes and male infertility in a large population of Chinese idiopathic infertile males [44]. Second, the folate concentration in the human blood plasma was not measured because previous studies showed that the blood folate level was not correlated with semen parameters but was positively associated with the seminal fluid folate [11, 18]. Finally, the methylation status of the *Esr1*, *Cav1*, and *Elavl1* promoters were not further detected in animal model. However, our study is the combination of clinical research and animal study. Moreover, the present study is one of the largest studies investigating the association of folate and semen parameters, presents reliable statistical evidence and eliminated strict inclusion and exclusion criteria.

In summary, our study highlights the importance of sufficient folate level in normal male reproductive function. Folate deficiency impaired spermatogenesis and reduced sperm concentration may partly due to inhibiting the expression of the three key molecules (*Esr1*, *Cav1* and *Elavl1*) essential for sperm production. In contrast to our speculation, the selected three genes may not be sensitive to methyl group deficiency; thus, their promoter methylation status was not altered. However, considering the effects of folate deficiency is complex and lasting, future research is needed to determine the significance of sufficient folate status on fertilization in men and subsequent pregnancy outcomes.

## MATERIALS AND METHODS

### Study subjects

**Figure 8:**
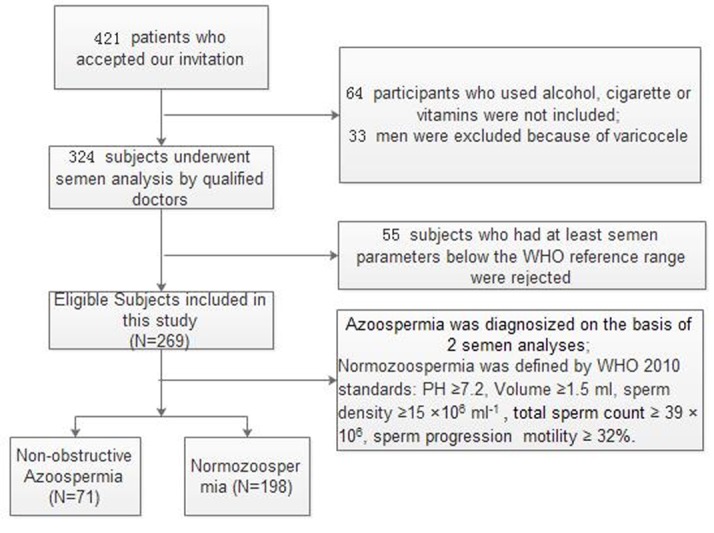
Flowchart of included men in this study

Male subfertile patients, aged 18 to 55 years, who attended the out-patient clinic of Wuhan Tongji Reproductive Medicine Hospital, were invited to participate in this study from March to August 2015. After the physical examination, all subjects were asked to fill out a questionnaire to collect the following data: age, medical history, body mass index (BMI), ethnicity, alcohol and cigarette use, abstinence length, and vitamin supplements. Participants who used alcohol, cigarettes, and vitamins or had varicocele were excluded in this study to avoid the effects of lifestyle factors, vitamin supplements and varicocele on semen parameters. Figure 8 presents the screening process of the study participants.

The study protocol was approved by the Reproductive Medicine Institutional Review Board of Tongji Medical College, Huazhong University of Science and Technology. All the participants provided an informed written consent after the doctors explained the study protocol. Clinical investigation was conducted in accordance with the principles expressed in the Declaration of Helsinki.

### Semen collection and analysis

After a recommended period of sexual abstinence (2-7 days), semen samples were collected by masturbation into a wide-mouthed sterile plastic container in a separate room. The ejaculates were marked with the subject's name and a serial number and immediately delivered to the laboratory. After liquefying in a water bath at 37 °C, a conventional semen analysis was conducted using a computer-assisted semen analysis system (SCA2000, Microptic, Barcelona, Spain) in accordance with the guidelines of the World Health Organization (2010). All samples were analyzed within 60 min by two well-trained laboratory technologists using the same apparatuses. During the research period, quality control was employed to ensure no significant difference between the results reported by the two laboratory operators.

### Determination of folate in seminal plasma

Immunoelectrochemoluminescence assay (Roche Modular E170; Roche Diagnostics GmbH, Mannheim, Germany) was used as previously described [21] to determine folate level in seminal plasma. The intra- and inter-assay coefficients of variation were 4.6% and 5.2%, respectively, at27 nmol/L. All samples were analyzed in duplicate in the same run.

### Animals and dietary treatments

Inbred C57BL/6 mice (female: 4 weeks old, n = 20; males: 5 weeks old, n = 10) were purchased from the Medical Experimental Animals of Hubei Province (Wuhan, China). All mice were housed in vivarium cages under standard conditions (22 °C and on a 12 L:12 D cycle). Experimental diets (folate deficient, FD) were purchased from Beijing HFK Bioscience Co. Ltd. (No.11003800008778). The dietary treatment was performed in accordance with the guidelines in the study of Lambrot [10]. Briefly, female mice were randomly divided into two groups. After 1 week of adaptation, mice in the folate-deficient sired group were fed with the FD diet (0.3 mg of folic acid per kilogram of body weight, n = 10) for 2 weeks and mated with non-experimental C57BL/6 males. The experimental group was maintained on the FD diet through pregnancy and lactation to generate folate-deficient males; from weaning at PND21, male pups in the next generation were also given the same experimental diet as their mothers until killing at 8 weeks. The control group and male mice were fed with regular mouse chow. Fresh water and food were provided ad libitum during the study period. Serum folate levels of mice were measured as previously described to verify the effectiveness of the mouse model [45]. At the time of killing, blood was collected into tubes containing EDTA, separated by centrifugation at 4000 × g for 7 min at 4 °C, and immediately examined using microbial assay. The testes tissues were kept in liquid nitrogen until assay. All animal procedures were authorized by the Animal Care and Use Committee of the Huazhong University of Science and Technology.

### Sperm counts and histopathological assays

Epididymal sperm count was determined as previously described [46]. Epididymis was collected from each group at the end of the 8th week and homogenized in 1 mL of saline. The homogenized samples were incubated at 37 °C under 5% CO_2_ saturated humidity for 30 min. Spermatozoa in 10 μL of the well-mixed sample were counted in the 25 squares of the chamber for four times. The testis samples were fixed, dehydrated, and embedded in paraffin. The tissue sections were cut into a thickness of 5 μm by using a Leica microtome and subjected to hematoxylin and eosin (HE) staining. After the slides were obtained, histopathological examination was performed and analyzed using the free software Image J.

### mRNA expression assay by real-time PCR

Total RNA was isolated from the two tissues by using TRIzol reagent (Invitrogen, CA, USA) in accordance with the protocols presented by Georgiadis [47] and Chen [48] to assay the gene expression of *ESR1, CAV1* and *ELAVL1* in the human sperm and mice testis. RNA was quantified, and its purity was assessed with Nanodrop 2000 spectrophotometer (Thermo Fisher Scientific, Waltham, MA, USA). RNA integrity was assessed by 2% agarose gel electrophoresis. RNA was treated with 1 unit of DNase I (Life Technologies, Maryland, USA) for 15 min at room temperature to eliminate DNA contamination. Reverse transcription was performed in accordance with the instruction of the first-strand cDNA synthesis kit (Fermentas, Toronto, Canada). Supplemental Table S2 shows the specific primers for *Esr1*, *Cav1*, *Elavl1*, and β-actin. Quantitative PCR analysis was conducted on KAPA SYBR FAST qPCR Kit Master Mix (2X) ABI Prism^TM^ (KAPA, KR0390). A 20 μL reaction volume containing 10 μL of 2 X Master Mix, 0.4 μL of each primer (10 pmol/μL), 2.5 μL of the template DNA, and 6.7 μL of ddH_2_O was placed in the RT-PCR apparatus. Analysis was performed under the following amplification conditions: initial denaturation at 95 °C for 3 min; followed by 40 cycles at 95 °C for 3 s, 60 °C for 30 s, and 72 °C for 60 s; and final elongation at 72 °C for 5 min. For appropriate negative controls, the RNA template was replaced with nuclease-free water, and amplification reactions were performed for each sample in triplicate. Relative mRNA expression was calculated using 2^−ΔΔCT^ method.

### Gene expression detection by western blot analysis

Western blot analysis was performed to determine the expression levels of*ESR1*, *CAV1*, and *ELAVL1*in testis tissues of mice from the experimental and control groups. Proteins were extracted from 200 mg of frozen testis tissues through homogenization and lysis in extraction buffer (100 μg/mL PMSE and RIPA). Protein concentration was determined using a Nanodrop 2000 spectrophotometer (Thermo Fisher, Waltham, MA, USA) through BCA method, followed by 12% sodium dodecyl sulfate-polyacrylamide gel electrophoretic separation. The protein was electrotransferred onto a polyvinylidene fluoride membrane, which was blocked by using a blocking buffer containing 20 mM Tris-HCl (pH 7.4), 150 mM NaCl, and 0.1% (v/v) Tween 20 (TTBS) plus 5% fat-free milk (w/v) for 1 h. The sample was incubated with a primary antibody (1:1,000; Earthox, Millbrae, CA, USA, Cat# E030130) at 4 °C overnight and with a secondary antibody (horseradish peroxidase-labeled goat anti-rabbit or goat anti-rat antibody 1:10,000) for 2 h. Results were obtained using the Enhanced Chemiluminescence (ECL) kit. Photographs were captured using a gel image analysis system. ESR1, CAV1, and ELAVL1 expression levels were normalized to that of the β-actin protein.

### Bisulfite sequencing (BSP) for gene promoter DNA methylation

Human sperm DNA was prepared through an optimized method described in our previous study [49]. Briefly, 2 μg of genomic DNA was treated with EpiTect Bisulfite Kit (Cat. No.59104, Qiagen) to assay DNA methylation of the *Esr1*, *Cav1*, and *Elavl1* promoters in the human sperm and mouse testis. Bisulfited DNA amplification was performed using a PCR instrument (Life Technologies, Grand Island, NY) through bisulfite sequencing. The BSP primers were designed by MethPrimer and listed in Supplemental Table S3. The PCR products were isolated with 2 % agarose gel electrophoresis and purified using a DNA gel extraction kit (Beyotime technology, Shanghai, China). The purified DNA was cloned into the PMD18-T vector (Takara, Dalian, China). Twenty positive clones from each sample were randomly selected for sequencing. DNA methylation levels were calculated based on the percentage of the methylated CpG sites divided by the total CpG sites in promoter region.

### Statistical analysis

All data were analyzed using Statistical Package for the Social Sciences (SPSS) software (version 17.0, SPSS, Inc., IL, USA). Results were expressed as median (range) or mean ± SD as appropriate. Mann-Whitney U test and Spearman rank correlation coefficients were used to determine the differences between groups and the associations between the semen parameters and total folate in seminal plasma, respectively, because of the skewed distribution of the data. Multiple linear regression analysis was further conducted to adjust for the effect of possible confounding variables on semen quality. The differences in the serum folate levels and the expression of *ESR1, CAV1* and *ELAVL1*were analyzed between groups by using independent student's t-test. For all tests, the differences among the values were considered significant at *P* < 0.05

## SUPPLEMENTARY MATERIALS TABLES


