# Supplementary material for: Effect of folate deficiency on promoter methylation and gene expression of Esr1, Cav1, and Elavl1, and its influence on spermatogenesis
Source: Oncotarget. 2017 Feb 25;8(15):24130–41. doi: 10.18632/oncotarget.15731 (PMC5421833; doi:10.18632/oncotarget.15731)
Supplement: Supplementary file 1 [file oncotarget-08-24130-s001.pdf]

Effect of folate deficiency on promoter methylation and gene expression of *Esr1*, *Cav1*, and *Elavl1*, and its influence on spermatogenesis

Supplementary Material

Supplemental Table S1: Characteristics of 20 subjects for RT-PCR and BSP assay.

Characteristic	Low folate (n= 10)[a]	High folate (n=10)[a]	*P*-value[b]
Seminal plasma folate	15.83 (11.27—17.08)	43.07 (40.44—43.66)	0.00
Demographic			
Age (y)	31 (25—39)	31 (26—40)	0.83
BMI (kg/m^2)	21.6 (19.6—26.0)	22.1 (18.4—27.5)	0.77
Semen parameters			
Ejaculate volume (mL)	4.4 (2.5—6.0)	3.4 (2.0—9.0)	0.47
Sperm density (10^6/mL)	55.87 (19.07—91.44)	182.11 (97.03—227.08)	0.00
Sperm count (x 10^6)	240.31 (61.01—394.40)	514.57 (279.63—1998.09)	0.02
Sperm progressive motility (%)	46.84 (32.39—65.37)	46.77 (35.98—64.13)	0.96
Sperm normal morphology (%)	43.84 (32.33—61.23)	46.34 (28.94—50.24)	0.81
Duration of abstention (d)	5 (3—7)	3 (2—7)	0.15

[a]All variables were given as median (range).
[b]independent student's t-test was used to determine the difference.

Supplemental Table S2: List of PCR primers

Sample	Gene	Forward Primer	Reverse Primer	Length of Product (bp)
Human sperm	*Esr1*	GCCAACGCGCAGGTCTA	GCCGCAGCCTCAGA	62
	Cav1	GCGACCCTAAACACCTCAAC	ATGCCGTCAAAACTGTGTGTC	91
	Elavl1	TGAACTACGTGACCGCGAAG	CCCAAACCGAGAGAACATGTC	194
	β-actin	GTCCACCGCAAATGCTTCTA	TGCTGTCACCTTCACCGTTC	190
Testis of mice	*Esr1*	GTCGCCTAGCCCGCTGAT	CATGTTTCCTTTCTCGTTACTGC	214
	Cav1	TAAATCACAGCCCAGGGAAAC	TGAGATGCTTGGGGTCGC	232
	Elavl1	AGGTTCCTCCGAGCCCATC	GCGTCTTGCCCAAGGTTGT	246
	β-actin	CTGAGAGGGAAATCGTGCGT	CCACAGGATTCCATACCCAAGA	208

Supplemental Table S3: List of BSP primers

Sample	Gene	Forward Primer	Reverse Primer	Length of Product (bp)
Human sperm	*Esr1*	AGATTAGTATTTAAAGTTGGAGGTT	ATATAAAAAATCATAATCATAATCC	283
	Cav1	TTTAAGAATTTTTTTTGTATTTTGG	TATCCTAAAACTCAATCTCACCTAC	206
	Elavl1	TTTGTGGGATTTTTYGGGGGA	ACGAACCRATATAAACRCCRA	164
